# Full-Length Genome Sequences of Recombinant and Nonrecombinant Sympatric Strains of *Rice yellow mottle virus* from Western Kenya

**DOI:** 10.1128/genomeA.01508-17

**Published:** 2018-02-22

**Authors:** Antony Kigaru Adego, Nils Poulicard, Agnès Pinel-Galzi, Benard Mukoye, Denis Fargette, Hassan Karakacha Wéré, Eugénie Hébrard

**Affiliations:** a Department of Biological Sciences, Masinde Muliro University of Science and Technology (MMUST), Kakamega, Kenya; b Interactions Plantes-Microorganismes-Environnement (IPME), Institut de Recherche pour le Développement (IRD), CIRAD, Université Montpellier, Montpellier, France

## Abstract

Five isolates of *Rice yellow mottle virus* from western Kenya were fully sequenced. One isolate of strain S4lv had been collected in 1966. Two isolates belonged to the emerging strain S4ug recently described in Uganda. Two isolates collected in 2012 are putative recombinants between the S4lv and S4ug strains.

## GENOME ANNOUNCEMENT

Rice yellow mottle virus (RYMV) is a major biotic constraint for rice cultivation in Africa, causing high losses in rice production (1). RYMV is a member of the genus Sobemovirus (2), and its genome consists of a single-stranded positive-sense RNA with five open reading frames (3). RYMV was classified in six major strains with marked spatial distribution (4). The highest diversity of the virus is found in East Africa, the putative center of origin of RYMV (5).

Recent surveys in new geographical areas highlighted the extent of recombination in RYMV evolution (6, 7). However, the epidemiological impact of these recombination events cannot be assessed, either because the putative recombinants and their “major” parents are not located within the same geographical areas or because the “minor” parents (i.e., the donors of the recombinant part) have not been identified. Here, we provide the first information on these decisive points by sequencing the complete genomes of five isolates from western Kenya. Isolate Ke2 had been collected in 1966 at Kisumu, close to Lake Victoria (8). Four isolates were collected in 2012 and 2016 in the same area (Kisumu County).

The sequences of the five isolates from Kenya were compared to the 23 published full-length sequences from East Africa using Molecular Evolutionary Genetics Analysis version 6.06 (MEGA) (9) and Recombination Detection Program version 4.94 (RDP4) (10). Phylogenetic studies confirmed that isolate Ke2 belongs to the strain S4lv found south of Lake Victoria, from Rwanda to southwest Kenya through northern Tanzania. Two isolates (Ke323 and Ke345) genetically close to each other (97.2% nucleotide identity) belong to the strain S4ug recently described in eastern Uganda (6) and in northern Tanzania (97.4% to 98.6%) (11, 12). The last two isolates (Ke101 and Ke105), also collected near Kisumu, are genetically close to each other (99.2%).

Recombination analyses indicated that isolates Ke101 and Ke105 are putative recombinants between strains S4lv and S4ug. They are genetically very close to isolate Ke2 (97.2%) and to other isolates of the strain S4lv of this region (96.4% to 96.9%), except in the C-terminal domain of the capsid protein (CP) and in the 3'-noncoding domain, which are more related to isolates Ke323 and Ke345 of the S4ug strain (97.2% to 97.8%). This is the first evidence of recombination within the coat protein gene for RYMV. Genetically, it is the first recombination event where both putative parents of a recombinant strain are identified. Epidemiologically, it is the first description of a recombinant strain and its putative parents that shared the same distribution area. Altogether, these results suggest the recent emergence of a new RYMV strain by recombination between sympatric strains.

### Accession number(s).

These genomic sequences for isolates Ke2, Ke101, Ke105, Ke323, and Ke345 have been deposited in GenBank under the accession no. MG599276 to MG599280, respectively.
